# Factors associated with carrying out activities to stimulate child development in the family context

**DOI:** 10.1016/j.jped.2026.101555

**Published:** 2026-06-01

**Authors:** Júlia Hannah Teixeira, Juliana Araújo Teixeira, Sonia Isoyama Venancio, Maria Beatriz Martins Linhares, Rogério Lerner, Naercio Aquino Menezes-Filho, Débora Falleiros de Mello, CPAPI - The Brazilian Center for Early Child Development – Centro Brasileiro de Pesquisa Aplicado à Primeira Infância

**Affiliations:** a Universidade de São Paulo, Escola de Enfermagem de Ribeirão Preto, Ribeirão Preto, SP, Brazil; b Universidade de São Paulo, Faculdade de Saúde Pública, São Paulo, SP, Brazil; c Instituto de Saúde da Secretaria de Saúde do Estado de São Paulo, São Paulo, SP, Brazil; d Universidade de São Paulo, Faculdade de Medicina de Ribeirão Preto, Ribeirão Preto, SP, Brazil; e Universidade de São Paulo, Instituto de Psicologia, São Paulo, SP, Brazil; f INSPER, São Paulo, SP, Brazil; g Universidade de São Paulo, Departamento de Economia, São Paulo, SP, Brazil

**Keywords:** Child development, Parent-child relations, Child care, Child health

## Abstract

**Objective:**

Early childhood stimulation is pivotal for enabling children to achieve their full developmental potential and thrive across the life course. Drawing on the Nurturing Care Framework (NCF) and the multigenerational life course model, this study examined the determinants of child development (CD) stimulation activities.

**Methods:**

This cross-sectional study was conducted with 365 mothers of children up to three years old who were seen at Primary Health Care units in a municipality in southeastern Brazil. Poisson regression with robust variance was performed to assess associations between contextual and environmental variables and the NCF domains and the practice of four or more stimulus activities, and to evaluate each activity individually.

**Results:**

Engaging in four or more stimulating activities was more prevalent among children who had books at home (IRR: 1.53; 95% CI: 1.23–1.92), attended daycare (IRR: 1.42; 95% CI: 1.15–1.75), and played with toys and/or household objects (IRR: 1.24; 95% CI: 1.02–1.51). The multigenerational life course model demonstrated that book ownership, daycare attendance, playing with toys and/or household objects, information received from health professionals, maternal occupation, and concern for CD were associated with engaging in activities that promote CD.

**Conclusion:**

The availability of books, toys, and play equipment in the home environment, regular attendance at daycare, and health guidelines to promote development make a difference in the first three years of life. The findings provide insights for public policies aimed at strengthening responsive care and learning opportunities for healthy early childhood.

## Introduction

Early childhood development (ECD) is one of the cornerstones of health, well-being, and productivity throughout life [1]. The first years of life are crucial moments in human development, building the foundation for the formation and refinement of complex skills [2]. Investing in ECD is therefore essential, especially in countries like Brazil, marked by diversity and profound social inequalities [3]. In the Brazilian context, approximately 12% of children in major state capitals show suspected developmental delays, a prevalence closely linked to family vulnerability, food insecurity, and low maternal education [4]. This scenario is aggravated by a growing number of Brazilian children at risk of being developmentally off-track, while significant gaps in information regarding stimulation in the home environment still persist [5].

Given the need to promote interventions related to ECD, scientific evidence underpinned the development of the Nurturing Care Framework (NCF) [6]. The NCF proposes five essential components to promote ECD: good health, adequate nutrition, safety and protection, responsive care, and learning opportunities from the beginning of life [2,6]. Beyond providing a structured model for child development (CD), this framework builds resilience, as stimulating environments buffer stressors like poverty and violences^1^. Within this framework, parental involvement in stimulating activities - such as reading, playing, and singing - promotes bonding, responsive care, and timely learning [7]. While joint caregiver stimulation is highly effective, [8] these practices remain inadequate in many low- and middle-income countries (LMICs) [9].

Complementing the role of shared caregiving, women’s empowerment is a strategic determinant for CD. Greater female agency, particularly in social independence, is consistently associated with better literacy, numeracy, and cognitive outcomes [10]. Empowered women can better provide essential nutrition and learning inputs, helping children reach their developmental potential [11]. Addressing these gaps is crucial for empowering families and achieving the Sustainable Development Goals (SDGs), especially in vulnerable populations where socioeconomic constraints often limit access to the resources necessary for healthy human development [12].

Previous Brazilian studies have focused on socioeconomic and environmental predictors of developmental outcomes, with few addressing daily stimulation practices at home [4,13]. Evidence shows that caregiver engagement in at least four stimulating activities with the child in the three days preceding the interview was consistently associated with a greater likelihood of being developmentally on track, both among children under 36 months and those aged 36-59 months [4]. This study makes a unique contribution by addressing these gaps, providing an integrated analysis of factors related to the NCF and demonstrating how their interaction shapes the nurturing environment in an upper- and middle-income country context.

The hypothesis is that both socioeconomic characteristics and the NCF domains influence the practice of stimulation activities by family members. Specifically, families living in a favorable socioeconomic context, with better access to health, nutrition, safety, responsive care, and learning opportunities, engage in stimulation activities more frequently compared to those facing conditions that are more adverse and gaps in these domains. Thus, the objective of this study is to identify factors associated with stimulation activities for child development in the family environment, in light of the NCF domains.

## Methods

### Study design and population

This is a cross-sectional study derived from a quasi-experimental before-and-after pilot study aimed at enhancing developmental surveillance using the Child Health Handbook in a municipality in southeastern Brazil. The analysis included 365 mothers and children under three years old across six Primary Health Care (PHC) units. The estimated population is 54,865 inhabitants and, in 2010 - the last year for which data is available - showed a Municipal Human Development Index of 0.777. In 2021, the per capita income was R$ 385,773.53, and in 2023, the infant mortality rate was 8.31 deaths per thousand live births [14]. The selection of the municipality took into account the local organization of PHC and the interest of municipal managers in the research.

The intervention involved training PHC professionals through distance learning and face-to-face workshops focused on surveillance of child development. Data for the present analysis were obtained from structured interviews with caregivers at two distinct time points: prior to the professional training (baseline) and following its implementation (endline). Intervention status (pre- vs. post-intervention) was included as a covariate in all multivariate statistical models to adjust for potential differences between the two data collection periods.

The sample size calculation was performed considering a standard deviation of 0.5, a power of 80%, and an alpha of 5%, resulting in 199 children before and 199 children after the intervention. The distribution of the sample among the six health units followed the proportion of pediatric consultations performed in each unit. The study initially included 412 maternal caregivers. Focusing exclusively on mothers ensured standardized responses and reduced reporting bias arising from differing perceptions among different types of caregivers. Children between 0 and 36 months old, residing in the studied municipality and under follow-up care at health units, were eligible. After applying these eligibility criteria, the sample consisted of 370 participants. Given that missing data represented only 1.3% of this group (n = 5) and power was not compromised, no statistical imputation was performed. A complete case analysis (listwise deletion) was adopted, as the impact of missing data at this level is considered negligible [15,16]. This resulted in a final analytical sample of 365 maternal caregiver-child pairs (175 interviewed before and 190 after the intervention). Although the final analytic sample was slightly smaller than the originally planned sample, the reduction was modest, and the higher prevalence of the secondary outcome (child development stimulation activities) ensured adequate precision of the estimates in the multivariable models.

Participants were selected during health unit visits for walk-in care, vaccinations, or scheduled appointments. Trained interviewers through individual face-to-face interviews using tablets, and carried out data collection. The fieldwork was carried out between December 2022 and April 2023 (before the intervention) and between November 2023 and March 2024 (after the intervention).

### Theoretical-conceptual model

**Fig. 1:**
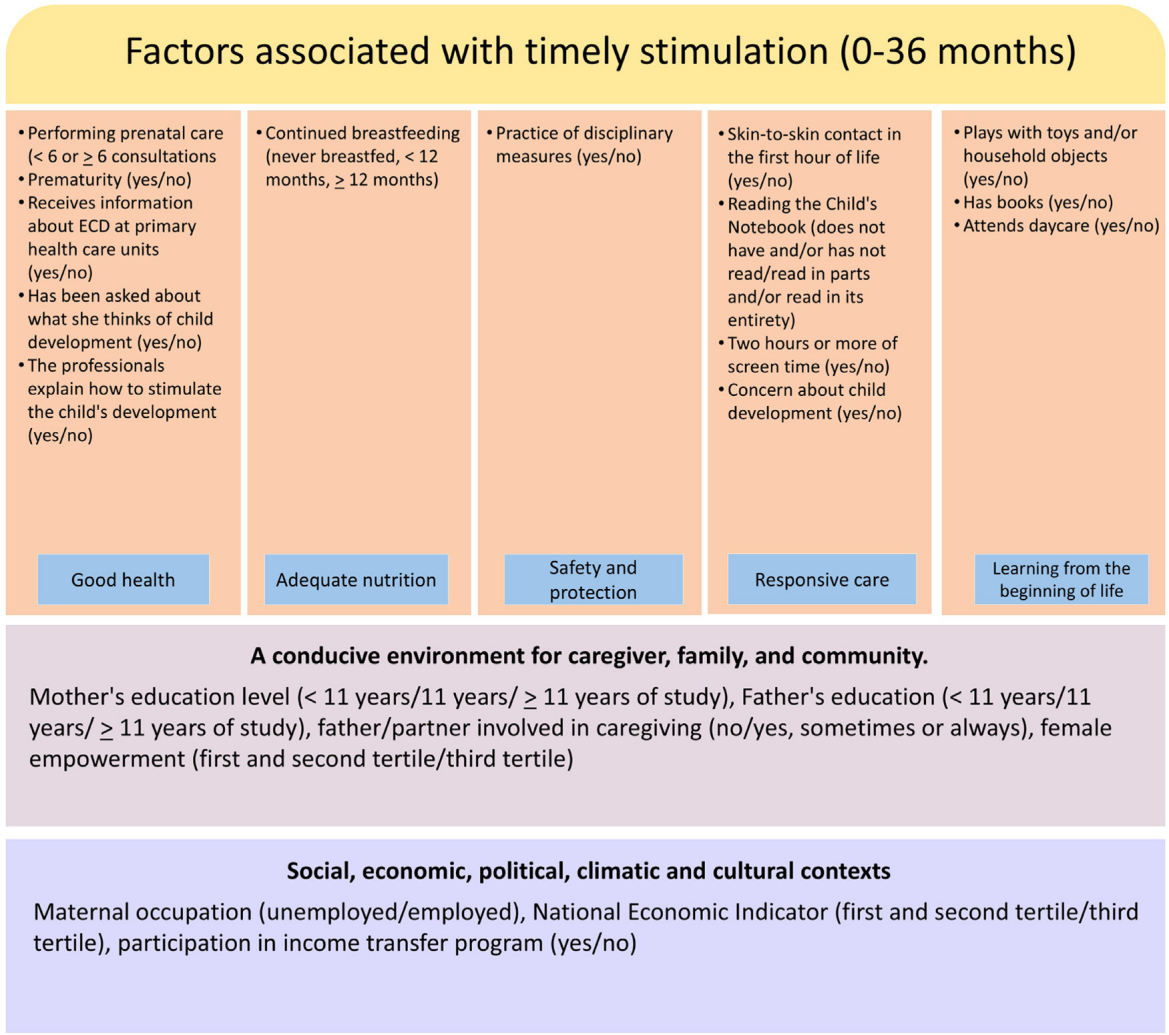
Conceptual model of contexts, environment and nurturing adapted from the multigenerational life course framework of early childhood development by Black et al.^1^

Source: modified from Black et al.^1^

Analysis was guided by an adapted life course conceptual framework for ECD^1^, to identify factors associated with stimulation activities across hierarchical levels (Figure 1).

### Outcome variable

The outcome variable was the performance of activities to stimulate CD in the family environment, considering the three days prior to the interview. This measure was based on Multiple Indicator Cluster Surveys-MICS [17] starting from the following question: “*In the last three days, have you or any other family member aged 15 or older been involved in any of the following activities with the child: Reading books or looking at pictures in books? Telling stories? Singing songs, including lullabies? Taking for a walk? Playing? Naming, counting, or drawing?* The possible answers were “yes”, “no”, or “don't know”. The variable was coded considering four or more stimulus activities as the cutoff point, [17] classified as “yes” or “no”; and each of the activities was evaluated individually, also as “yes” or “no”.

### Exposure variables

The questions were based on the QAD-PIPAS, a quick and low-cost instrument for CD monitoring in Brazilian vaccination campaigns [18]. The questions were organized by the five NCF domains and adapted from international tools like MICS [17] and the National Economic Indicator (IEN) [19]. Data collection occurred before appointments, limiting supplementary instruments.

Exposure variables were organized into three hierarchical levels (Supplementary Material S1). The distal level represents the socioeconomic, political, and cultural context in which the child and their family are embedded. The intermediate level corresponds to the family environment and parental conditions, including maternal and paternal education and the father’s participation in caregiving, a variable that represents the dimension of gender equity. Women’s social independence was also considered one of the domains of the Survey-based Women’s emPowERment Index-SWPER, [10] which incorporated frequency of reading newspapers or magazines, years of schooling completed, maternal age at first childbirth, age at first cohabitation with a partner, and age and educational differences between the woman and her partner [10]. The proximal level was represented by variables directly related to the NCF domains [2,6]. The domain of safety and protection included disciplinary parenting practices such as yelling, spanking, and hitting the child.

### Data analysis

Data were analyzed using descriptive and analytical statistics, including absolute and relative frequencies with 95% confidence intervals (95% CI). Differences in proportions according to stimulus activities (< 4 lt; 4 vs. ≥ 4) were evaluated using chi-square or Fisher’s exact tests.

To assess the association between carrying out stimulating activities and the exposure variables, Poisson regression models with robust variance were used to estimate incidence rate ratios (IRR) and their respective 95% confidence intervals (CI). The analyses were conducted for each activity individually and for the categorized outcome. For the adjusted model, all predictor variables listed in the theoretical model were included simultaneously due to their conceptual relevance in explaining the outcome. Moreover, the models were adjusted for child’s sex (female or male), child’s age (0-12, 13-24, and 25-36 months), and the timing of data collection (pre-intervention and post-intervention).

The significance level adopted was α < 0.05, and the analyses were performed using Stata software, version 16 [20].

### Ethical aspects

The study was approved by the Research Ethics Committee of the Institute of Health of the State Secretariat of Health of São Paulo (CAAE: 61653622.4.0000.5469). All participating maternal caregivers provided informed consent.

## Results

### Sample characteristics

Most of the mothers were between 20 and 34 years old (71%), had 11 years of schooling (61%), and were employed (60.6%). Among paternal caregivers, the same age range (62.2%) and education level (53.4%) prevailed, with maternal reports indicating that a large proportion of them were involved in the child's care (87.5%). Regarding socioeconomic conditions, 30.3% of caregivers reported food insecurity, and approximately 19% participated in income transfer programs. With regard to women's empowerment, the majority were found in the least empowered groups (67%). Among the children, more than half were female (53.9%), white (52.6%), and aged between 0 and 12 months (63.3%). Furthermore, approximately 45% did not have siblings living in the same house, and the majority did not attend daycare or preschool (79.5%).

Table 1 presents the characterization of the sample according to the exposure variables of the theoretical model and the outcome variable.

**Table 1:** Characterization of the sample according to the exposure variables of the theoretical model and the outcome variable, 2025.

DISTAL BLOCK							
Predictor variables	TOTAL		< 4 ACTIVITIES		≥ 4 ACTIVITIES		p-value^b^
	n	%	n	%	n	%	
**Maternal occupation**							
Employed^a^	220	60.6	86	60.6	134	60.6	0.99
Unemployed	143	39.4	56	39.4	87	39.4	
**National Economic Indicator**							0.09
First and second tercile	234	66.7	98	72.1	136	63.3	
Third tercile	117	33.3	38	27.9	79	36.7	
**Participation in an income transfer program**							0.50
No	292	81.1	116	82.9	176	80.0	
Yes	68	18.9	24	17.1	44	20.0	

INTERMEDIATE BLOCK							
Predictor variables	TOTAL		< 4 ACTIVITIES		≥ 4 ACTIVITIES		p-value^b^
	n	%	n	%	n	%	
**Mother's education level**							0.002
< 11 years	68	18.7	26	18.3	42	18.9	
11 years (Finished High School)	222	61.0	100	70.4	122	55.0	
> 11 years	74	20.3	16	11.3	58	26.1	
**Father's education level**							0.003
< 11 years	95	26.0	33	23.1	62	27.9	
11 years (Finished High School)	195	53.4	91	63.6	104	46.9	
> 11 years	75	20.6	19	13.3	56	25.2	
**The father/partner gets involved in the care.**							0.08
No	45	12.5	23	16.3	22	10.1	
Yes, sometimes / always	314	87.5	118	83.7	196	89.9	
**Women's Empowerment (SWPER)**							0.10
First and second tercile	209	67.0	87	72.5	122	63.5	
Third tercile	103	33,0	33	27,5	70	36,5	

PROXIMAL BLOCK							
*Good health*							
Predictor variables	TOTAL		< 4 ACTIVITIES		≥ 4 ACTIVITIES		p-value^b^
	n	%	n	%	n	%	
**Prenatal care**							1.00
< 6 consultations	8	2.2	3	2.1	5	2.3	
≥ 6 consultations	350	97.8	139	97.9	211	97.7	
**The child was born prematurely.**							0.07
No	329	90.1	134	93.7	195	87.8	
Yes	36	9.9	9	6.3	27	12.2	
**Receives information about ECD at primary health care units.**							0.04
No	106	29.1	50	35.2	56	25.2	
Yes	258	70.9	92	64.8	166	74.8	
**She has already been asked what she thinks about child development.**							0.03
No	199	54.7	88	61.5	111	50.2	
Yes	165	45.3	55	38.5	110	49.8	
**The professionals explain how to stimulate the child's development.**							0.005
No	100	27.5	51	35.7	49	22.2	
Yes	264	72.5	92	64.3	172	77.8	

*Proper nutrition*							
Predictor variable	TOTAL		< 4 ACTIVITIES		≥ 4 ACTIVITIES		p-value^b^
	n	%	n	%	n	%	
**Continued breastfeeding**							<0.001
Never breastfed	21	5.8	8	5.6	13	5.9	
< 12 months	265	72.6	123	86.0	142	64.0	
≥12 months	79	21.6	12	8.4	67	30.2	

*Safety/protection*							
Predictor variable	TOTAL		< 4 ACTIVITIES		≥ 4 ACTIVITIES		p-value^b^
	n	%	n	%	n	%	
**Disciplinary parenting practices^c^**							0.003
No	269	74.9	116	83.5	153	69.6	
Yes	90	25.1	23	16.6	67	30.5	

*Responsive care*							
Predictor variables	TOTAL		< 4 ACTIVITIES		≥ 4 ACTIVITIES		p-value^b^
	n	%	n	%	n	%	
**Skin-to-skin contact in the first hour of life.**							0.73
No	66	18.1	27	19.0	39	17.6	
Yes	298	81.9	115	81.0	183	82.4	
**Reading from the Child's Register**							0.37
Does not have / Has not read	163	44.7	68	47.6	95	42.8	
Read parts / All	202	55.3	75	52.5	127	57.2	
**Two hours or more of screen time**							0.76
No	334	91.8	132	92.3	202	91.4	
Yes	30	8.2	11	7.7	19	8.6	
**Concern for the child's development**							0.74
No	314	87.2	124	87.9	190	86.8	
Yes	46	12.8	17	12.1	29	13.2	

*Learning from the beginning of life*							
Predictor variables	TOTAL		< 4 ACTIVITIES		≥ 4 ACTIVITIES		p-value^b^
	n	%	n	%	n	%	
**Plays with toys and/or household objects**^d^							<0.001
No	201	55.1	106	74.1	95	42.8	
Yes	164	44.9	37	25.9	127	57.2	
**Has books**							<0.001
No	181	49.7	107	74.8	74	33.5	
Yes	183	50.3	36	25.2	147	66.5	
**Attends daycare.**							<0.001
No	290	79.5	131	91.6	159	71.6	
Yes	75	20.6	12	8.4	63	28.4	

Control variables	TOTAL		< 4 ACTIVITIES		≥ 4 ACTIVITIES		p-value^b^
	n	%	n	%	n	%	
**Child's sex**							0.13
Male	168	46.2	73	51.1	95	43.0	
Female	196	53.9	70	49.0	126	57.0	
**Child's age**							<0.001
0-12 months	231	63.3	115	80.4	116	52.3	
13-24 months	90	24.7	21	14.7	69	31.1	
25-36 months	44	12.1	7	4.9	37	16.7	
**Data collection**							0.93
Pre-intervention	175	48.0	69	48.3	106	47.8	
Post-intervention	190	52.1	74	51.8	116	52.3	

The differences observed in the totals compared to the reference (n) were due to “missing” (no information). Missing: maternal occupation (n = 2), national economic indicator (n = 14), participation in income transfer program (n = 5), mother's education (n = 1), father/partner involvement in care (n = 6), female empowerment (n = 53), prenatal care (n = 7), receiving information about DPI in primary health care units (n = 1), being asked about her opinion regarding child development (n = 1), professionals explaining how to stimulate child development (n = 1), disciplinary parenting practices (n = 6), skin-to-skin contact in the first hour of life (n = 1), two hours or more of screen time (n = 1), concern about child development (n = 5), owning books (n = 1) and child's sex (n = 1).

(a) Formal employment (n = 164), self-employed (n = 44), informal (n = 12).

(b) Fisher's Exact Test for values < 5.

(c) Disciplinary parenting practices considered in the analysis were yelling, slapping, and hitting the child.

(d) The child plays with homemade toys, store-bought toys, and/or household objects.

### Factors associated with carrying out four or more activities to stimulate child development in the family context

Owning books (IRR: 1.53, 95% CI: 1.23-1.92), attending daycare (IRR: 1.42, 95% CI: 1.15-1.75), and playing with toys and/or household objects (IRR: 1.24, 95% CI: 1.02-1.51) increased the prevalence of engaging in activities that stimulate CD by 53%, 42%, and 24%, respectively, compared to children without these factors (Table 2). Additionally, paternal education of 11 years showed a counterintuitive association with the outcome, being related to an 18% lower prevalence of engaging in activities that stimulate CD (IRR: 0.82; 95% CI: 0.67-0.99), compared to children whose fathers had less than 11 years of schooling.

**Table 2:** Robust variance Poisson regression analysis, with adjustment, of the association between covariates and performance of each stimulation activity, 2025.

DISTAL BLOCK			
Predictor variables	≥ 4 ACTIVITIES TO STIMULATE CD		
	IRR	CI (95%)	p-value
**Maternal occupation**			
Employed^a^	1		
Unemployed	1.15	(0.94; 1.41)	0.18
**National Economic Indicator**			
First and second tercile	1		
Third tercile	0.94	(0.74; 1.19)	0.59
**Participation in an income transfer program**			
No	1		
Yes	1.22	(0.97; 1.54)	0.09

INTERMEDIATE BLOCK			
Predictor variables	≥ 4 ACTIVITIES TO STIMULATE CD		
	IRR	CI (95%)	p-value
**Mother's education level**			
< 11 years	1		
11 years (Finished High School)	0.92	(0.71; 1.20)	0.55
> 11 years	1.11	(0.78; 1.56)	0.57
**Father's education level**			
< 11 years	1		
11 years	0.82	(0.67; 0.99)	0.04
> 11 years	1.05	(0.80; 1.39)	0.71
**The father/partner gets involved in the care.**			
No	1		
Yes, sometimes / always	1.26	(0.89; 1.80)	0.20
**Women's Empowerment (SWPER)**			
First and second tercile	1		
Third tercile	1.03	(0.82; 1.29)	0.81

PROXIMAL BLOCK			
*Good Health*			
Predictor variables	≥ 4 ACTIVITIES TO STIMULATE CD		
	IRR	CI (95%)	p-value
**Prenatal care**			
< 6 consultations	1		
≥ 6 consultations	0.78	(0.35; 1.75)	0.55
**The child was born prematurely.**			
No	1		
Yes	1.26	(0.90; 1.75)	0.18
**Receives information about ECD at primary health care units.**			
No	1		
Yes	1.24	(0.96; 1.59)	0.10
**She has already been asked what she thinks about child development.**			
No	1		
Yes	0.96	(0.80; 1.16)	0.67
**The professionals explain how to stimulate the child's development.**			
No	1		
Yes	1.01	(0.81; 1.26)	0.94

*Proper nutrition*			
Predictor variable	≥ 4 ACTIVITIES TO STIMULATE CD		
	IRR	CI (95%)	p-value
**Continued breastfeeding**			
Never breastfed	1		
< 12 months	0.86	(0.54; 1.37)	0.53
≥ 12 months	1.09	(0.69; 1.73)	0.70

*Safety/protection*			
Predictor variable	≥ 4 ACTIVITIES TO STIMULATE CD		
	IRR	CI (95%)	p-value
**Disciplinary parenting practices^b^**			
No	1		
Yes	1.03	(0.85; 1.24)	0.80

*Responsive care*			
Predictor variables	≥ 4 ACTIVITIES TO STIMULATE CD		
	IRR	CI (95%)	p-value
**Skin-to-skin contact in the first hour of life.**			
No	1		
Yes	1.06	(0.83; 1.36)	0.64
**Reading from the Child's Register**			
Does not have / Has not read	1		
Read parts / All	1.10	(0.91; 1.32)	0.32
**Two hours or more of screen time**			
No	1		
Yes	1.00	(0.73; 1.36)	0.98
**Concern for the child's development**			
No	1		
Yes	0.94	(0.74; 1.19)	0.60

*Learning from the beginning of life*			
Predictor variables	≥ 4 ACTIVITIES TO STIMULATE CD		
	IRR	CI (95%)	p-value
**Plays with toys and/or household objects^c^**			
No	1		
Yes	1.24	(1.02; 1.51)	0.03
**Has books**			
No	1		
Yes	1.53	(1.23; 1.92)	<0.001
**Attends daycare.**			
No	1		
Yes	1.42	(1.15; 1.75)	0.001

Control variables	≥ 4 ACTIVITIES TO STIMULATE CD		
	IRR	CI (95%)	p-value
**Child's sex**			
Male	1		
Female	1.15	(0.95; 1.39)	0.15
**Child's age**			
0-12 months	1		
13-24 months	0.99	(0.78; 1.25)	0.93
25-36 months	0.93	(0.69; 1.25)	0.62
**Data collection**			
Pre-intervention	1		
Post-intervention	0.98	(0.82; 1.17)	0.85

(a) Formal employment (n = 164), self-employed (n = 44), informal (n = 12).

(b) Disciplinary parenting practices considered in the analysis were yelling, slapping, and hitting the child.

(c) The child plays with homemade toys, store-bought toys, and/or household objects.

IRR, Incidence Rate Ratio - adjusted for child's sex, child's age, and data collection period (pre/post-intervention).

95%CI, Confidence Interval.

The unadjusted analysis of the associations between exposure variables and the performance of activities that stimulate CD is available in Supplementary Material S2.

### Factors associated to each of the stimulus activities

Considering the context of the children, maternal occupation was associated with play activity, with unemployed mothers showing a 10% higher prevalence of this practice compared to those with employment (IRR: 1.10; 95% CI: 1.00-1.21) (Table 3). There was no association between environmental variables and the different stimulus activities.

**Table 3:** Robust variance Poisson regression analysis, with adjustment, of the association between covariates and performance of each stimulation activity, 2025.

DISTAL BLOCK																		
Predictor variables	ACTIVITIES TO STIMULATE CD																	
	Reading			Telling a story			Singing			Strolling			Playing			Naming / Counting / Drawing		
	IRR	CI (95%)	p-value	IRR	CI (95%)	p- value	IRR	CI (95%)	p- value	IRR	CI (95%)	p- value	IRR	CI (95%)	p- value	IRR	CI (95%)	p- value
**Maternal occupation**																		
Employed^a^	1			1			1			1			1			1		
Unemployed	1.07	(0.80; 1.43)	0.66	1.09	(0.83; 1.42)	0.54	0.98	(0.91; 1.07)	0.71	1.05	(0.96; 1.14)	0.30	1.10	(1.00; 1.21)	0.04	1.34	(1.00;1.81)	0.054
**National Economic Indicator**																		
First and second tercile	1			1			1			1			1			1		
Third tercile	0.87	(0.63; 1.19)	0.39	0.86	(0.63; 1.17)	0.34	0.98	(0.88; 1.09)	0.71	0.99	(0.88; 1.11)	0.87	1.04	(0.93; 1.15)	0.48	0.84	(0.61; 1.14)	0.26
**Participation in an income transfer program**																		
No	1			1			1			1			1			1		
Yes	1.13	(0.80; 1.59)	0.50	1.12	(0.82; 1.55)	0.48	1.06	(0.94; 1.19)	0.33	1.10	(0.99; 1.21)	0.07	1.06	(0.94; 1.20)	0.32	1.14	(0.84; 1.56)	0.40

INTERMEDIATE BLOCK																		
Predictor variables	ACTIVITIES TO STIMULATE CD																	
	Reading			Telling a story			Singing			Strolling			Playing			Naming / Counting / Drawing		
	IRR	CI (95%)	p- value	IRR	CI (95%)	p- value	IRR	CI (95%)	p- value	IRR	CI (95%)	p- value	IRR	CI (95%)	p- value	IRR	CI (95%)	p- value
**Mother's education level**																		
< 11 years	1			1			1			1			1			1		
11 years (Finished High School)	0.96	(0.67; 1.38)	0.82	0.79	(0.57; 1.10)	0.17	1.08	(0.94; 1.24)	0.27	0.97	(0.88; 1.08)	0.60	0.99	(0.88; 1.12)	0.91	0.75	(0.51; 1.09)	0.13
> 11 years	1.36	(0.87; 2.15)	0.18	1.16	(0.76; 1.78)	0.48	1.14	(0.94; 1.39)	0.19	0.94	(0.81; 1.10)	0.46	1.13	(0.94; 1.35)	0.20	1.10	(0.70; 1.74)	0.67
**Father's education level**																		
< 11 years	1			1			1			1			1			1		
11 years (Finished High School)	0.82	(0.62; 1.09)	0.17	0.87	(0.66; 1.14)	0.32	0.94	(0.85; 1.03)	0.18	0.97	(0.88; 1.06)	0.44	0.99	(0.90; 1.09)	0.81	0.86	(0.65; 1.15)	0.31
> 11 years	1.00	(0.66;1.51)	0.98	1.24	(0.85; 1.82)	0.27	0.97	(0.85; 1.11)	0.64	1.01	(0.87; 1.18)	0.87	1.01	(0.89; 1.16)	0.84	1.03	(0.66; 1.59)	0.90
**The father/partner gets involved in the care.**																		
No	1			1			1			1			1			1		
Yes, sometimes / always	1.26	(0.80; 1.98)	0.32	1.20	(0.79; 1.82)	0.39	1.10	(0.91; 1.32)	0.34	1.00	(0.87; 1.15)	0.99	1.09	(0.94; 1.28)	0.26	1.14	(0.71; 1.83)	0.59
**Women's empowerment**																		
First and second tercile	1			1			1			1			1			1		
Third tercile	0.95	(0.71; 1.27)	0.74	0.90	(0.66; 1.22)	0.49	1.02	(0.91; 1.14)	0.77	1.06	(0.96; 1.17)	0.25	0.88	(0.77; 1.02)	0.09	0.96	(0.70; 1.30)	0.79

PROXIMAL BLOCK																		
*Good health*																		
Predictor variables	ACTIVITIES TO STIMULATE CD																	
	Reading			Telling a story			Singing			Strolling			Playing			Naming / Counting / Drawing		
	IRR	CI (95%)	p- value	IRR	CI (95%)	p- value	IRR	CI (95%)	p- value	IRR	CI (95%)	p- value	IRR	CI (95%)	p- value	IRR	CI (95%)	p- value
**Prenatal care**																		
< 6 consultations	1			1			1			1			1			1		
≥ 6 consultations	0.87	(0.14; 5.26)	0.88	0.93	(0.31; 2.83)	0.91	0.79	(0.68; 0.92)	0.002	0.84	(0.72; 0.97)	0.02	0.92	(0.79; 1.06)	0.25	0.83	(0.26; 2.65)	0.75
**The child was born prematurely.**																		
No	1			1			1			1			1			1		
Yes	1.48	(0.95; 2.29)	0.08	1.44	(0.94; 2.21)	0.09	1.05	(0.91; 1.21)	0.50	1.08	(0.97; 1.21)	0.18	1.01	(0.86; 1.19)	0.89	1.04	(0.66; 1.63)	0.87
**Receives information about ECD at primary health care units.**																		
No	1			1			1			1			1			1		
Yes	1.53	(1.04; 2.26)	0.03	1.28	(0.87; 1.89)	0.21	1.06	(0.94; 1.20)	0.34	1.10	(0.98; 1.22)	0.10	1.10	(0.97; 1.26)	0.15	1.28	(0.91; 1.81)	0.16
**She has already been asked what she thinks about child development.**																		
No	1			1			1			1			1			1		
Yes	0.96	(0.75; 1.24)	0.77	1.11	(0.87; 1.42)	0.39	0.96	(0.88; 1.04)	0.32	1.00	(0.93; 1.08)	0.96	0.98	(0.90; 1.07)	0.73	0.82	(0.61; 1.10)	0.19
**The professionals explain how to stimulate the child's development.**																		
No	1			1			1			1			1			1		
Yes	1.02	(0.74; 1.40)	0.91	1.13	(0.82; 1.55)	0.45	1.07	(0.96; 1.20)	0.20	0.97	(0.89; 1.06)	0.46	1.05	(0.94; 1.18)	0.37	1.12	(0.80;1.55)	0.51

*Proper nutrition*																		
Predictor variables	ACTIVITIES TO STIMULATE CD																	
	Reading			Telling a story			Singing			Strolling			Playing			Naming / Counting / Drawing		
	IRR	CI (95%)	p- value	IRR	CI (95%)	p- value	IRR	CI (95%)	p- value	IRR	CI (95%)	p- value	IRR	CI (95%)	p- value	IRR	CI (95%)	p- value
**Continued breastfeeding**																		
Never breastfed	1			1			1			1			1			1		
< 12 months	1.29	(0.59; 2.82)	0.52	1.00	(0.52; 1.91)	0.99	0.95	(0.83; 1.10)	0.48	1.02	(0.85; 1.21)	0.85	0.93	(0.81; 1.06)	0.27	0.89	(0.52; 1.55)	0.69
≥ 12 months	1.93	(0.89; 4.19)	0.10	1.15	(0.60; 2.18)	0.68	0.92	(0.77; 1.11)	0.39	1.10	(0.91; 1.32)	0.32	0.95	(0.82; 1.10)	0.48	1.08	(0.65; 1.81)	0.76

*Safety/protection*																		
Predictor variables	ACTIVITIES TO STIMULATE CD																	
	Reading			Telling a story			Singing			Strolling			Playing			Naming / Counting / Drawing		
	IRR	CI (95%)	p- value	IRR	CI (95%)	p- value	IRR	CI (95%)	p- value	IRR	CI (95%)	p- value	IRR	CI (95%)	p- value	IRR	CI (95%)	p- value
**Disciplinary parenting practices^b^**																		
No	1			1			1			1			1			1		
Yes	1.03	(0.78; 1.37)	0.82	1.17	(0.90; 1.50)	0.24	0.97	(0.87; 1.07)	0.49	0.95	(0.86; 1.05)	0.29	0.96	(0.88; 1.06)	0.45	0.90	(0.67; 1.20)	0.47

*Responsive care*																		
Predictor variables	Reading			Telling a story			Singing			Strolling			Playing			Naming / Counting / Drawing		
	IRR	CI (95%)	p- value	IRR	CI (95%)	p- value	IRR	CI (95%)	p- value	IRR	CI (95%)	p- value	IRR	CI (95%)	p- value	IRR	CI (95%)	p- value
**Skin-to-skin contact in the first hour of life.**																		
No	1			1			1			1			1			1		
Yes	1.31	(0.91; 1.87)	0.15	0.98	(0.71; 1.35)	0.89	1.03	(0.90; 1.18)	0.65	0.98	(0.91; 1.06)	0.66	1.00	(0.89; 1.12)	0.97	0.97	(0.70; 1.33)	0.83
**Reading from the Child's Register**																		
Does not have / Has not read	1			1			1			1			1			1		
Read parts / All	1.11	(0.86; 1.43)	0.42	1.06	(0.83; 1.35)	0.62	0.96	(0.89; 1.04)	0.36	0.98	(0.91; 1.05)	0.56	1.00	(0.90; 1.10)	0.93	0.94	(0.72; 1.23)	0.65
**Two hours or more of screen time**																		
No	1			1			1			1			1			1		
Yes	0.70	(0.43; 1.14)	0.15	0.90	(0.55; 1.46)	0.67	0.97	(0.81; 1.17)	0.76	1.02	(0.89; 1.18)	0.77	1.09	(0.97; 1.21)	0.15	1.03	(0.69; 1.55)	0.87
**Concern for the child's development**																		
No	1			1			1			1			1			1		
Yes	1.00	(0.73; 1.37)	0.98	0.97	(0.70; 1.34)	0.85	1.07	(0.99; 1.17)	0.09	1.02	(0.92; 1.12)	0.74	1.10	(1.01; 1.21)	0.03	1.11	(0,81; 1,52)	0,52

*Learning from the beginning of life*																		
Predictor variables	Reading			Telling stories			Singing			Strolling			Playing			Naming / Counting / Drawing		
	IRR	CI (95%)	p- value	IRR	CI (95%)	p- value	IRR	CI (95%)	p- value	IRR	CI (95%)	p- value	IRR	CI (95%)	p- value	IRR	CI (95%)	p- value
**Plays with toys and/or household objects^c^**																		
No	1			1			1			1			1			1		
Yes	1.28	(0.98; 1.66)	0.07	1.55	(1.16; 2.06)	0.003	1.05	(0.96; 1.14)	0.31	1.08	(1.00; 1.16)	0.049	1.11	(1.03; 1.20)	0.006	1.22	(0.91; 1.63)	0.18
**Has books**																		
No	1			1			1			1			1			1		
Yes	2.08	(1.46; 2.96)	0.000	1.51	(1.12; 2.04)	0.006	1.14	(1.04; 1.25)	0.003	1.09	(1.00; 1.19)	0.06	1.01	(0.92; 1.10)	0.91	1.66	(1.16; 2.36)	0.005
**Attends daycare.**																		
No	1			1			1			1			1			1		
Yes	1.73	(1.28; 2.33)	0.000	1.17	(0.87; 1.56)	0.31	1.04	(0.93; 1.16)	0.50	1.03	(0.94; 1.13)	0.50	1.14	(1.04; 1.25)	0.005	1.67	(1.23; 2.26)	0.001

Control variables	ACTIVITIES TO STIMULATE CD																	
	Reading			Telling stories			Singing			Strolling			Playing			Naming / Counting / Drawing		
	IRR	CI (95%)	p- value	IRR	CI (95%)	p- value	IRR	CI (95%)	p- value	IRR	CI (95%)	p- value	IRR	CI (95%)	p- value	IRR	CI (95%)	p- value
**Child's sex**																		
Male	1			1			1			1			1			1		
Female	1.36	(1.03; 1.79)	0.03	1.15	(0.90; 1.46)	0.26	0.99	(0.92; 1.07)	0.83	1.02	(0.95; 1.10)	0.62	1.04	(0.95; 1.14)	0.36	1.09	(0.83; 1.43)	0.56
**Child's age**																		
0-12 months	1			1			1			1			1			1		
13-24 months	0.92	(0.64; 1.34)	0.67	0.94	(0.69; 1.28)	0.68	0.97	(0.87; 1.08)	0.55	0.93	(0.84; 1.03)	0.16	1.07	(0.99; 1.16)	0.11	1.36	(0.92; 2.00)	0.12
25-36 months	0.84	(0.54; 1.31)	0.440	1.07	(0.73; 1.56)	0.73	0.88	(0.75; 1.02)	0.08	0.90	(0.77; 1.03)	0.13	1.01	(0.90; 1.12)	0.92	1.60	(1.03; 2.47)	0.04
**Data collection**																		
Pre-intervention	1			1			1			1			1			1		
Post-intervention	0.71	(0.55; 0.91)	0.01	1.12	(0.87; 1.45)	0.37	1.08	(1.00; 1.17)	0.06	1.00	(0.93; 1.07)	0.98	1.14	(1.04; 1.25)	0.005	0.84	(0.64; 1.10)	0.22

(a) Formal employment (n = 164), self-employed (n = 44), informal (n = 12).

(b) Disciplinary parenting practices considered in the analysis were yelling, slapping, and hitting the child.

(c) The child plays with homemade toys, store-bought toys, and/or household objects.

IRR, Incidence Rate Ratio - adjusted for child's sex, child's age, and data collection period (pre/post-intervention).

95%CI, Confidence Interval.

In the proximal block, having six or more prenatal visits was associated with a lower prevalence of singing (IRR: 0.79; 95% CI: 0.68-0.92) and taking a walk (IRR: 0.84; 95% CI: 0.72-0.97). Receiving information about CD at health units increased the prevalence of reading by 53% (IRR: 1.53; 95% CI: 1.04-2.26), demonstrating the relationship between the NCF domain, good health, and stimulation activities. Maternal concern about CD increased the prevalence of play by 10% (IRR: 1.10; 95% CI: 1.01-1.21), related to the domain of *responsive care*.

In the domain of learning from the beginning of life*,* playing with toys and/or household objects was associated with a higher prevalence of storytelling (IRR: 1.55 95% CI: 1.16-2.06), playing (IRR: 1.11 95% CI: 1.03-1.20), and going for a walk (IRR: 1.08 95% CI: 1.00-1.16). Owning books was positively associated with most activities, with a notable 108% increase in reading (IRR: 2.08, 95% CI: 1.46-2.96). Attending daycare was associated with a higher prevalence of reading (IRR: 1.73 95% CI: 1.28-2.33), naming, counting and drawing (IRR: 1.67 95% CI: 1.23-2.26), and playing (IRR: 1.14 95% CI: 1.04-1.25).

## Discussion

This study identified that proximal factors related to early life learning are strongly associated with engaging in stimulation activities. The factors most associated with engaging in at least four stimulating activities at home were playing with toys and/or household objects, having books, and attending daycare. These findings advance the literature by demonstrating how specific proximal factors - aligned with the NCF domains - outweigh distal socioeconomic constraints in predicting high-frequency stimulation. Access to resources and opportunities, such as receiving information from PHC professionals and maternal concern for CD, highlights the specificity of our study by reinforcing that the PHC setting is a powerful entry point for promoting responsive care and early learning, particularly in contexts where social inequalities might otherwise limit these opportunities.

Our findings reinforce that child stimulation is not an isolated act but part of a broader caregiving environment. The presence of toys and books reflects a parental propensity for engagement, [21] aligning with the responsive caregiving domain of the NFC. The authors advance this understanding by demonstrating that caregiver engagement is often intertwined with formal support systems, such as daycare centers. While previous studies have emphasized the direct benefits of daycare on cognitive and socio-emotional outcomes through early learning opportunities, [22,23] our findings suggest that these effects extend beyond the institutional setting. Specifically, daycare enrollment may reflect proactive caregiving or actively shape family practices through increased interaction between institutions and families, as recommended by national guidelines [24]. This potential bidirectional influence reinforces the idea that early childhood education can indirectly enhance the quality of the home environment, thereby amplifying its impact on CD.

In this study, information on CD received in PHC was a strong predictor of home reading activities. This reinforces the role of healthcare professionals in expanding caregiver knowledge, particularly during childcare consultations [25]. PHC teams must be prepared to guide families on the importance of early childhood stimulation. In line with evidence that proactive counseling and the provision of reading materials by pediatricians improve the quality of parent-child interactions, [26] our data highlight the need to capacitate PHC professionals for consistent developmental guidance. Strengthening their educational role is a critical public health strategy to promote nurturing care and early literacy within the community.

Unexpectedly, maternal caregivers who attended more than six prenatal appointments showed a lower prevalence of singing and taking walks with the child. While prenatal care is a primary entry point for the good health domain of the NCF, our results suggest that frequent clinical contact does not automatically translate into the promotion of responsive caregiving. Professional interventions during pregnancy, aimed at promoting mother/father-baby interaction, are fundamental to strengthening parental self-efficacy and emotional bonds [27]. Thus, the result found deserves investigation in future research that considers mediating and moderating variables capable of influencing the relationship between the number of prenatal visits and practices that encourage CD.

Paternal engagement remains a critical gap in the responsive caregiving and opportunities for early learning domains. Our findings reflect a persistent gender disparity, similar to other studies [28,29] where paternal involvement was not significantly associated with stimulation practices, likely reinforced by traditional roles that centralize childcare as a maternal responsibility. Notably, our study reveals an unexpected inverse association between higher paternal education (> 11 gt; 11 years) and stimulation activities. While higher education typically correlates with better developmental outcomes [30] this discrepancy suggests that unmeasured factors, such as the type of occupation and working hours, may limit fathers’ interaction time. Furthermore, since only mothers provided data, potential response bias must be considered. These results emphasize the need for PHC professionals to transcend maternal-centered approaches and implement father-inclusive strategies to dismantle gender-based barriers to nurturing care.

This study has some limitations. The use of a non-probabilistic convenience sample requires caution in generalizing the findings to other contexts. Data on stimulation activities relied on maternal self-report regarding the previous three days, which is susceptible to recall bias and social desirability bias, potentially leading to an overestimation of practices. The practice of stimulating activities was considered through maternal self-report and, therefore, may not encompass the entire reality of other caregivers, and paternal participation in care may have been underreported. As it has a cross-sectional design, with variable association, it was not possible to establish a causal relationship between the variables. Furthermore, although the study included all health units in the municipality to ensure representativeness, formal cluster-adjusted modeling was not the primary approach. However, sensitivity analyses including the health unit as a covariate demonstrated no significant changes in the magnitude or direction of the associations, suggesting that inter-unit variance did not bias the results. This is likely supported by the standardized nature of local health protocols across the municipal network. Finally, some variables that could be related to the outcome were not considered because their frequency in the sample was low, such as maternal depression.

A key strength of this study is its practical contribution to early childhood interventions within a real-world primary care setting. Our findings demonstrate that home resources - toys and books - and daycare attendance are major drivers of parental stimulation practices. Variables related to early life learning proved to be more decisive in increasing the prevalence of four or more stimulating activities. Furthermore, receiving developmental information from PHC professionals directly impacted reading practices, reinforcing the centrality of PHC in promoting nurturing care. The NCF-aligned structure, integrated with the life course model, highlights the essential domains and interventions needed to strengthen early childhood policies.

Future research should include paternal involvement, broader age ranges, and longitudinal designs to better understand childcare dynamics and stimulation practices, ultimately supporting the reduction of inequalities and healthy child development.

## Authors’ contributions

M.Sc. Júlia Teixeira performed the analyses, wrote the initial version of the manuscript, and revised and edited the manuscript.

Dr. Juliana Teixeira conceived and designed the study, coordinated and supervised data collection, and revised and edited the manuscript.

Dr. Sonia Venancio conceived and designed the study, coordinated and supervised data collection, and revised and edited the manuscript.

Dr. Maria Beatriz Linhares conceived and designed the study, collaborated in the data discussion, and revised and edited the manuscript.

Dr. Rogério Lerner conceived and designed the study, collaborated in the data discussion, and revised and edited the manuscript.

Dr. Naercio Menezes-Filho conceived and designed the study, collaborated in the data discussion, and revised and edited the manuscript.

Dr. Débora F. Mello conceived and designed the study, coordinated and supervised data collection, and revised and edited the manuscript.

The Brazilian Center for Early Child Development coordinated data collection, and coordinated funding.

All authors approved the final version of the manuscript as submitted and agreed to be responsible for all aspects of the work.

## Financial support

This study was financed by São Paulo Research Foundation (FAPESP), Brasil. Process Number 2024/01218-4.

## Declaration of generative AI and AI-assisted technologies in the manuscript preparation process

During the preparation of this work, the authors used Gemini in order to improve language and readability. After using this tool/service, the authors reviewed and edited the content as needed and take full responsibility for the content of the published article.

## Data availability statement

The data that support the findings of this study are available from the corresponding author upon reasonable request.

## Conflicts of interest

The authors declare no conflicts of interest.

## Appendix Supplementary materials

Image, application 1
