# Supplementary material for: Factors associated with carrying out activities to stimulate child development in the family context
Source: J Pediatr (Rio J). 2026 Jun 1;102(4):101555. doi: 10.1016/j.jped.2026.101555 (PMC13251659; doi:10.1016/j.jped.2026.101555)
Supplement: Supplementary file 1 [file mmc1.docx]

**JPED-D-25-00557_ Supplementary Material**

**Supplementary Material S1** Strategy for grouping the data collected in the study for each level of the conceptual framework of CD - CPAPI - Brazil, 2025.

| **Conceptual model** | **Variables** | **Themes represented** |
| --- | --- | --- |
| **Level 1 (distal) - Social, economic, political, climatic and cultural contexts** | | |
| Government support to the family and poverty reduction; Maternal employment; Housing security and conditions; Reduction of extreme weather events; Sanitation and drinking water; Political commitment regarding parental leave and guidelines for childcare support; Social safety nets. | Maternal occupation | Socioeconomic context |
|  | National Economic Indicator (IEN) |  |
|  | Income transfer programs | Political commitment to supporting the family |
| **Level 2 (intermediate) - Environment conducive to caregiver, family and community.** | | |
| Education of parental caregivers; Mental and physical health of parental caregivers; Age at marriage; Nutrition and care during pregnancy and childbirth; Spacing between births; Family planning; Safe neighborhood; Gender equality; Absence of stigma/prejudice. | Mother's education level | Maternal and paternal educational level |
|  | Father's education level |  |
|  | Father's involvement in childcare | Gender equality |
|  | How often women read newspapers or magazines. | Women's empowerment (social independence) |
|  | Women's education in years of completed schooling |  |
|  | Woman's age at first childbirth |  |
|  | Woman's age at first cohabitation |  |
|  | Age difference: woman's age minus husband's age. |  |
|  | Educational gap: woman's years of schooling minus husband's years of schooling. |  |
| **Level 3 (proximal) - Nurturing care: Good health** | | |
| Prenatal care; Birth conditions; Childcare follow-up; Immunizations; Disease prevention and treatment; Promotion of health and human development. | Prenatal care | Health and CD Promotion |
|  | Prematurity |  |
|  | Information about ECD offered in PHC |  |
| **Level 3 (proximal) - Nurturing care: Proper nutrition** | | |
| Breastfeeding; Dietary diversity; Healthy complementary feeding. | Continued breastfeeding | Child nutrition |
| **Level 3 (proximal) - Nurturing care: Safety and protection** | | |
| Birth registration; Prevention of mistreatment and reduction of adversity (abuse, neglect, violence); Early intervention for vulnerable children (e.g., disabled, malnourished, orphans); Healthy environments and safe recreational spaces. | Disciplinary parenting practices (yelling, spanking, and hitting) | Adversities in childhood |
| **Level 3 (proximal) - Nurturing care: Responsive care** | | |
| Care routines; Responsive parenting; Home visits and encouragement of positive parenting; Parenting programs and promotion of human development. | Skin-to-skin contact in the first hour of life. | Responsive parenting |
|  | Reading from the Child's Register |  |
|  | Two hours or more of screen time |  |
|  | Concern about CD |  |
| **Level 3 (proximal) - Nurturing care: Learning from the beginning of life** | | |
| Opportunities at home to stimulate language, exploration, and learning; the presence and use of age-appropriate books, toys, materials, and games; access to quality childcare, preschool, and school. | Has toys. | Stimulating children's cognitive development |
|  | Has books |  |
|  | Attends daycare or preschool | Primary education |

| **Supplementary material S2** Robust variance Poisson regression analysis, with no adjustment, of the association between covariates and performance of 4 or more stimulation activities, 2025. | | | | | |
| --- | --- | --- | --- | --- | --- |
| **DISTAL BLOCK** | | | | | |
| **Predictor variables** | **≥ 4 ACTIVITIES TO STIMULATE CD** | | | |  |
|  | IRR | (95%CI) | p value | | model p-value |
| **Maternal occupation** |  |  |  | |  |
| Employed^a^ | 1 |  |  | | 0.99 |
| Unemployed | 1.00 | (0.84; 1.18) | 0.99 | |  |
| **National Economic Indicator** |  |  |  | |  |
| First and second tercile | 1 |  |  | | 0.08 |
| Third tercile | 1.16 | (0.98; 1.37) | 0.08 | |  |
| **Participation in an income transfer program** |  |  |  | |  |
| No | 1 |  |  | | 0.48 |
| Yes | 1.07 | (0.88; 1.31) | 0.49 | |  |
| **INTERMEDIATE BLOCK** | | | | | |
| **Predictor variables** | **≥ 4 ACTIVITIES TO STIMULATE CD** | | | |  |
|  | IRR | (95%CI) | p value | | model p-value |
| **Mother's education level** |  |  |  | |  |
| < 11 years | 1 |  |  | | <0.001 |
| 11 years | 0.89 | (0.71; 1.11) | 0.30 | |  |
| > 11 years | 1.27 | (1.02; 1.58) | 0.04 | |  |
| **Father's education level** |  |  |  | |  |
| < 11 years | 1 |  |  | |  |
| 11 years | 0.82 | (0.67; 1.00) | 0.045 | | 0.002 |
| > 11 years | 1.14 | (0.94; 1.39) | 0.18 | |  |
| **The father/partner gets involved in the care.** |  |  |  | |  |
| No | 1 |  |  | | 0.12 |
| Yes, sometimes / always | 1.28 | (0,94; 1,74) | 0,12 | |  |
| **Women's empowerment** |  |  |  | |  |
| First and second tercile | 1 |  |  | | 0,09 |
| Third tercile | 1.16 | (0,98; 1,39) | 0,09 | |  |
| **PROXIMAL BLOCK** | | | | | |
| ***Good health*** | | | | | |
| **Predictor variables** | **≥ 4 ACTIVITIES TO STIMULATE CD** | | | | |
|  | IRR | (IC95%) | p value | | model p-value |
| **Prenatal care** |  |  |  | |  |
| < 6 consultations | 1 |  |  | | 0,90 |
| ≥ 6 consultations | 0.96 | (0.56; 1.66) | 0.90 | |  |
| **The child was born prematurely.** |  |  |  | |  |
| No | 1 |  |  | | 0.03 |
| Yes | 1.27 | (1.03; 1.56) | 0.03 | |  |
| **Receives information about ECD at primary health care units.** |  |  |  | |  |
| No | 1 |  |  | | 0.06 |
| Yes | 1.22 | (1.00; 1.49) | 0.06 | |  |
| **She has already been asked what she thinks about child development.** |  |  |  | |  |
| No | 1 |  |  | | 0.03 |
| Yes | 1.20 | (1.01; 1.41) | 0.03 | |  |
| **The professionals explain how to stimulate the child's development.** |  |  |  | |  |
| No | 1 |  |  | | 0.01 |
| Yes | 1.33 | (1.07; 1.65) | 0.01 | |  |
| ***Adequate nutrition*** | | | | | |
| **Predictor variable** | **≥ 4 ACTIVITIES TO STIMULATE CD** | | | | |
|  | IRR | (95%CI) | p value | | model p-value |
| **Continued breastfeeding** |  |  |  | |  |
| Never breastfed | 1 |  |  | | <0.001 |
| < 12 months | 0.87 | (0,61; 1,23) | 0,43 | |  |
| ≥ 12 months | 1.37 | (0,97; 1,94) | 0,08 | |  |
| ***Safety and protection*** | | | | | |
|  | | | | | |
| **Predictor variable** | **≥ 4 ACTIVITIES TO STIMULATE CD** | | | | |
|  | IRR | (95%CI) | p value | | model p-value |
| **Disciplinary parenting practices^b^** |  |  |  | |  |
| No | 1 |  |  | | 0.001 |
| Yes | 1.31 | (1.12; 1.54) | 0.001 | |  |
| ***Responsive care*** | | | | | |
| **Predictor variables** | **≥ 4 ACTIVITIES TO STIMULATE CD** | | | | |
|  | IRR | (95%CI) | p value | | model p-value |
| **Skin-to-skin contact in the first hour of life.** |  |  |  | |  |
| No | 1 |  |  | | 0.73 |
| Yes | 1.04 | (0.83; 1.30) | 0.73 | |  |
| **Reading from the Child's Register** |  |  |  | |  |
| Does not have / Has not read | 1 |  |  | | 0.38 |
| Read parts / All | 1.08 | (0.91; 1.28) | 0.38 | |  |
| **Two hours or more of screen time** |  |  |  | |  |
| No | 1 |  |  | | 0.75 |
| Yes | 1.05 | (0.79; 1.39) | 0.75 | |  |
| **Concern for the child's development** |  |  |  | |  |
| No | 1 |  |  | | 0.74 |
| Yes | 1.04 | (0.82; 1.32) | 0.74 | |  |
| ***Learning from the beginning of life*** | | | | | |
| **Predictor variables** | **≥ 4 ACTIVITIES TO STIMULATE CD** | | | | |
|  | IRR | (95%CI) | p value | model p-value | |
| **Plays with toys and/or household objects^c^** |  |  |  | |  |
| No | 1 |  |  | | <0.001 |
| Yes | 1.64 | (1.39; 1.94) | <0.001 | |  |
| **Has books** |  |  |  | |  |
| No | 1 |  |  | | <0.001 |
| Yes | 1.96 | (1.63; 2.37) | <0.001 | |  |
| **Attends daycare.** |  |  |  | |  |
| No | 1 |  |  | | <0.001 |
| Yes | 1.53 | (1.33; 1.77) | <0.001 | |  |
| **Control variables** | **≥ 4 ACTIVITIES TO STIMULATE CD** | | | | |
|  | **IRR** | **CI (95%)** | **p value** | | **model p-value** |
| **Child's sex** |  |  |  | |  |
| Male | 1 |  |  | | 0.14 |
| Female | 1.14 | (0.96; 1.35) | 0.14 | |  |
| **Child's age** |  |  |  | |  |
| 0-12 months | 1 |  |  | |  |
| 13-24 months | 1.53 | (1.29; 1.81) | <0.001 | | <0.001 |
| 25-36 months | 1.67 | (1.40; 2.01) | <0.001 | |  |
| **Data collection** |  |  |  | |  |
| Pre-intervention | 1 |  |  | | 0.93 |
| Post-intervention | 1.01 | (0.85; 1.19) | 0.93 | |  |
| (a) Formal employment (n = 164), self-employed (n = 44), informal (n = 12)  (b) Disciplinary parenting practices considered in the analysis were yelling, slapping, and hitting the child.  (c) The child plays with homemade toys, store-bought toys, and/or household objects.  IRR: Incidence Rate Ratio - adjusted for child's sex, child's age, and data collection period (pre/post-intervention)  95%CI Confidence Interval | | | | | |
